# Analyzing the Influence of Wine and Beer Drinking, Smoking, and Leisure Time Screen Viewing Activity on Body Weight: A Cross-Sectional Study in Germany

**DOI:** 10.3390/nu13103553

**Published:** 2021-10-11

**Authors:** Elena Raptou, Georgios Papastefanou

**Affiliations:** 1 Laboratory of Management and Marketing, Department of Agricultural Development, Democritus University of Thrace, 68200 Orestiada, Greece; 2 GESIS-Leibniz Institute for the Social Sciences, P. O. Box 122155, 68072 Mannheim, Germany; info@bodymonitor.de

**Keywords:** BMI, drinking, smoking, leisure time screen viewing behaviors, endogeneity, seemingly unrelated bivariate probit model, quantile regression

## Abstract

The increasing global prevalence of overweight and obesity highlights an urgent need to explore modifiable obesogenic factors. This study investigated the impact of lifestyle factors, such as beer and wine drinking, cigarette smoking, and leisure time screen viewing activities, on body weight and the development of obesity. Individual level data were selected from a random sample of 3471 German adults using a two-stage disproportionate random sampling procedure. The empirical analysis employed a two-stage equations system and combined the endogenous treatment effects model with the quantile regression technique. Our estimations showed that the decisions to smoke and consume wine and beer were positively interrelated, especially in women. Frequent beer/wine drinkers of normal weight were found to have a lower BMI in the male subsample. Quantile regression estimates indicated a significant influence of smoking on BMI in both genders, with smokers’ BMI following an upward trend, especially in the upper quantiles of the distribution. Leisure time screen activity was found to have a major impact on females’ BMI. Prolonged television viewing and regular computer gaming had a strong relationship with weight increase in overweight women, whereas internet surfing was inversely correlated with the BMI of normal weight and slightly overweight female participants. Nutrition and health policies should direct individuals toward alternative recreational activities in order to substitute screen usage and reduce sedentary time. This study also raised doubts about the general belief that smokers have a lower body weight. As unhealthy behaviors usually co-occur or cluster together, obesity prevention interventions might also contribute to a decrease in smoking.

## 1. Introduction

Today, obesity has reached epidemic proportions, with recent forecasts showing that 60% of the global population will be overweight or obese by 2030 [1,2]. This increasing trend in obesity rates is mainly attributed to environmental and cultural changes associated with a high energy density diet, the rise of a sedentary lifestyle, and the increased portion size perceived as the appropriate serving size [2,3,4].

Identifying the risk determinants for increased energy intake is of great importance to battle obesity. Alcohol drinking has been linked to a greater acute impact on calorie intake than other lifestyle factors [5] and alcoholic beverages rank among the top contributors to total energy intake [6]. Alcohol (ethanol) has a higher energy content per gram (29 kJ/g) compared with protein (17 kJ/g) or carbohydrates (16 kJ/g), and a slightly lower energy content than fats (37 kJ/g) [7]. There is evidence that moderate doses of alcohol (~0.6 g/kg) augment alcohol cravings and stimulate further drinking through biologic pathways, which may explain the correlation between alcohol intake and body weight [8]. Metabolic evidence shows that an increase in alcohol intake represses oxidation and increases body fat deposition, resulting in weight gain [8,9]. Alcohol consumption may arouse the neurotransmitter activity, causing an increase in alcohol’s positive reinforcement and reward, as well as stimulate the m-opioid system with opioid agonists, leading to a higher consumption of palatable foods [8,10,11]. Thus, alcohol drinking may not only be linked with a higher urge to drink, but also to less-restricted eating behavior. The association between alcohol consumption and body weight can also be explained by the diminished inhibitory mechanisms following drinking episodes, which may induce additional risky practices, such as overeating [8,12].

Although the high caloric content of most alcoholic drinks and the stimulatory effect of alcohol on food intake could trigger weight gain, recent reviews indicate an unclear cause-and-effect relationship between alcohol drinking and body weight and a lack of consistent evidence that drinking behavior is linked to weight increase [13]. A considerable body of literature noted a rather insignificant or a negative relationship between drinking behavior and body weight [14,15,16,17,18], whereas other studies encountered a positive correlation between drinking patterns and BMI [13,17]. These contradictory results may be attributed to differences in intake patterns and consumption frequencies. In particular, frequent light or moderate alcohol drinking seems to be less likely to contribute to weight gain than heavy drinking [13,19].

Beer and wine have a lower concentration of ethanol than spirits [20], and consumers seem to perceive that their moderate consumption may be related to positive health consequences [21,22]. Although there is a widespread belief that wine is healthier than beer [23] and beer consumption is supposed to be responsible for abdominal adiposity, published work does not support this perception when beer is consumed in moderation [24]. According to Silva et al., this general belief may be ascribed to the lack of information on the possible health benefits of moderate beer consumption, as most of the literature has focused on wine attributes [22]. Both wine and beer are associated with food as complementary to meals and are consumed in social contexts, mainly with friends or family in the home setting [22]. Therefore, their consumption might increase the likelihood of overeating and obesity risks, as a consequence of the social facilitation of eating [25].

Compounding the detrimental physiological effects of obesity, recent evidence has underlined a strong relationship between higher body weight and negative psychological consequences [26]. As weight increase is considered unfavorable, general fear of gaining weight may affect smoking behavior. The widespread perception that smoking may protect against obesity constitutes a motive for smoking initiation, whereas concerns about body image and body dissatisfaction are stronger in smokers than in non-smokers [27,28]. Weight concerns have also been suggested as a potential obstacle to smoking cessation for current smokers [29].

However, the relationship between smoking and obesity is complex and the published literature provides conflicting results. While an inverse relationship between smoking behavior and body weight has been highlighted in several studies [30,31,32,33], other studies have noted a rather insignificant correlation between smoking and BMI [34,35]. According to Baum et al. and Fang et al., this contradiction can be attributed to differences in model specification and/or the measurement of cigarette costs [30,31]. Given that smoking and obesity are associated with detrimental health effects and comprise cumulative risk factors for certain diseases, the concurrent development of both behaviors may augment health risks in some individuals. Consequently, there has been great interest in understanding the influence of cigarette smoking on body weight and the nature of their relationship.

As scientific evidence shows that a sedentary lifestyle contributes to an increase in obesity, there is accumulating evidence underlining the critical role of television viewing on body weight. Watching television (TV), as the predominant sedentary behavior, has been found to promote weight gain and result in negative health outcomes [4,36,37,38]. TV viewing may hamper individuals’ ability to react to internal hunger and satiety cues, and instead result in a strong dependence on external cues related to TV screen time [39]. Although watching TV during meal time is a common practice for many individuals, completing a meal while watching TV can result in increased energy intake and weight gain [40,41]. On the other hand, the number of studies that explored the influence of recreational internet and computer usage on BMI is limited in adult populations. Van Dyck et al. [42] and Thomée et al. [43] identified insignificant relationships between leisure time internet and BMI, whereas Vandelanotte et al. [44] found a strong association between high recreational internet time and overweight.

The present study sought to explore the influence of smoking and beer and wine drinking on individuals’ BMI after adjusting for various sociodemographic characteristics. Furthermore, the impact of leisure time screen activity on body weight was investigated using several indicators to distinguish among watching TV, surfing the internet, chatting and social networking, and computer gaming. To accomplish the aims of this study, we employed a two-stage equations system based of the endogenous treatment effects model and the quantile regression technique [35]. We also test for gender differences on the influence of the key variables on individuals’ body weight. Recent evidence showed noticeable differences in smoking and drinking behavior between men and women and a lower prevalence of both alcohol drinking and smoking in female participants [6,45]. Furthermore, women are more likely to engage in leisure time screen activity, especially watching TV, compared with their male counterparts [38].

## 2. Materials and Methods

### 2.1. Sample Selection

The individual level data adopted for this study were taken from the 2014 German General Social Survey (ALLBUS 2014) on social monitoring of trends in attitudes, behavior, and societal change in the Federal Republic of Germany. The survey was designed and accomplished by GESIS-Leibniz Institute for the Social Science. The sample selection methodology incorporated a two-stage disproportionate random sampling procedure in Western Germany (including West Berlin) and Eastern Germany (including East Berlin). The sample population included German and non-German adults who resided in private households. Targeted individuals who did not have an adequate background of the German language to conduct the interview were treated as systematic unit non-responses. The full data set was selected in September 2014 (six-month survey from 24 March 2014 to 13 September 2014).

The sample selection included a two-stage disproportionate random sampling procedure. A comprehensive explanation of GESIS survey guidelines for the two-stage sampling procedure is provided by Gabler and Hader [46]. In the first sample stage, municipalities (in both Western and Eastern Germany) were randomly selected with a probability proportional to their adult population size, ending up with 111 sample points in 103 Western Germany municipalities and 51 sample points in 45 Eastern Germany municipalities. In the second sample stage, respondents were also randomly selected from the municipal registers. Therefore, the random sampling techniques resulted in an adequate number of sample points to ensure a representative model of Germany’s settlement structure as well as a sample size representative of the total population.

Data were collected through in-person interviews with a standardized questionnaire and two additional self-completion questionnaires [47]. Participation was voluntary and the data selection procedure was approved by GESIS-Leibniz Institute of the Social Sciences. The present study was also given ethical approval by the Ethics Committee of Democritus University of Thrace. The response rate for the questionnaires completion was 35.0% and 35.1% for Western and Eastern Germany, respectively, resulting in the selection of 3471 valid questionnaires [48].

### 2.2. Measures

All participants self-reported their height and weight, and body mass index (BMI) defined as weight in kilograms divided by the square of height in meters (kg/m^2^) was recorded for indicating nutritional status [49]. For adults over 20 years old, the BMI cutoffs to describe normal weight, overweight, and obesity are 18.5, 25, and 30, respectively.

Survey participants were also asked about their current smoking behavior. The dichotomous smoking participation indicator took the value of 1 for smokers and 0 for non-smokers.

Furthermore, wine and beer consumption was delineated by a seven-point frequency scale ranging from several times a day to never (several times a day, every day/almost every day, several times a week, about once a week, twice or three times a month, once a month or less often, never). The seven categories of the scale were made into two categories with a “daily or almost daily” frequency to be the threshold for weekly frequent wine/beer consumption [50,51].

Leisure time screen activity included TV viewing and involvement with computer gaming and leisure time internet activities. Participants were asked how many days a week they watch TV, and their responses ranged from never to all seven days. The majority of participants (71.6%) reported that they watch TV on a daily basis. Furthermore, they were required to specify the average time spent watching TV on these days. A continuous variable was obtained to ascertain daily TV viewing time after multiplying the responses to the aforementioned questions and dividing by seven. TV viewing time was dichotomized as either high (at least 120 min/day) or low (less than 120 min/day) based on a median split [52,53]. Furthermore, participants were asked to point out the frequency of their involvement in (i) playing games on the computer, (ii) using the internet (surfing), and (iii) chatting and using social networks on the internet on a five-point scale ranging from every day to never (every day, at least once a week, at least once a month, less often, never). Three dichotomous indicators were constructed to describe regular (frequent) leisure time usage, taking the value of 1 for participants who had an involvement with the specific activity on a daily basis and 0 otherwise.

Respondents were asked to report the frequency of meeting their friends according to 1 of 5 categories ranging from every day to never (every day, at least once a week, at least once a month, less often, never). A frequency of at least once a week was suggested as the cut-off point to indicate frequent social interactions.

The socio-demographic profile of participants was described by various dichotomous variables, such as gender, age (18–29 years old, 30–60 years old, older than 60 years old), educational attainment (up to secondary educational level, post-secondary education including short cycle tertiary level and bachelor, postgraduate education including Master’s and PhD), marital status (divorced/widowed, married including registered partnership, single), and area of residence (big city, town, rural area/village).

### 2.3. Theoretical Rationale

There were some conditions that should be acknowledged. As smoking, alcohol drinking, and obesity are of public health concern, the existence of interactions between them is quite possible.

(1) First, endogeneity issues might affect smoking and drinking estimates. In case of endogeneity, the estimates of smoking and drinking will not represent their true effect on body weight. Endogeneity may be attributed to reverse causality (simultaneity), omitted variables, and measurement errors [31,54]. Recent evidence has established correlative links among body weight, weight management, and smoking. Individuals may provoke smoking behavior for weight control purposes, as there is a widespread perception that smokers have a lower body weight and smoking constitutes a weight control strategy, or vice versa [30,55]. With regard to drinking behavior, regular drinkers may be more body weight conscious than non-drinkers in order to compensate for the health impacts of drinking and the additional calorie intake of alcohol consumption, or vice versa.

Omitted variables bias may arise from unobserved person-specific factors. Therefore, there may be unobserved factors, such as depression and risk aversion, that may simultaneously affect both smoking behavior and body weight [56]. In addition, the relationship between alcohol drinking and body weight seems to be influenced by the potential of residual confounding by unmeasured variables that hamper the possibility to obtain unbiased estimates of drinking on weight [13]. For instance, sleeping habits have been shown to be correlated with both drinking patterns and body weight status [57]. Measurement errors may also contribute to endogeneity issues, as weight and height, smoking behavior, and frequency of wine and beer drinking were self-reported. Therefore, for all the above-mentioned reasons, this study had to correct for potential endogeneity.

(2) Second, potential interdependency between smoking and drinking behaviors should be considered. Witkiewitz et al. investigated concurrent smoking and alcohol drinking among young adults and showed that individuals drank more while smoking and consumed more cigarettes while drinking [58]. Furthermore, both decisions can be jointly determined by unobservable factors, such as depression and stress [13,59]. Therefore, ignoring potential reciprocity (simultaneity) between smoking and drinking could lead to serious estimation bias [35,60].

(3) Third, the influences of the main variables of interest (i.e., smoking, beer/wine drinking, leisure time screen activities) may differ across different segments of the BMI distribution (heterogeneity) [35]. Consequently, standard linear regressions (OLS) might lead to a statistical loss of information as they estimate the impact of various covariates on the conditional mean of the BMI, and may over- or underestimate the influence of the covariates at different points across the BMI distribution [61]. To deal with all the aforementioned conditions, this study employed a two-stage methodological approach, which is analytically described below.

### 2.4. Methodological Framework

To acknowledge for potential endogeneity issues and the heterogeneous effects of smoking, drinking, and leisure time screen activity on body weight, the empirical analysis employed the approach of Chang et al. [35]. Therefore, the econometric framework comprised a two-stage equations system and combined the features of the endogenous treatment effects model [62] and the quantile regression technique [63].

In the first stage, we explored the determinants of smoking behavior and wine/beer drinking, as well as the interrelationship between the two decisions. Potential simultaneity between smoking and frequent wine/beer drinking could lead to biased and inconsistent estimations [35,64]. To address this problem, we adopted a seemingly unrelated bivariate probit model approach, in which smoking participation and wine/beer consumption were simultaneously modelled and the correlation coefficient (*ρ)* estimated the degree of simultaneity between the two outcomes [65,66]. The equation describing the decision to smoke included various explanatory variables, such as age, marital status, area of residence, educational attainment, socializing, and the indicators describing leisure time screen viewing activity. The drinking equation encompassed a vector of explanatory variables depicting age, marital status, area of residence, socializing, and leisure time screen viewing indicators. As education is strongly linked to health knowledge, it is supposed to have more of an impact on smoking than beer and wine drinking. Socializing indicator was also encompassed in the seemingly unrelated bivariate probit model, as both smoking and wine/beer drinking are, to some extent, influenced by peers or friends [22,35].

In the second stage, two important conditions had to be considered before constructing the body weight equation. The first condition referred to potential endogeneity between (i) smoking and body weight and (ii) frequent wine/beer drinking and body weight. In line with the endogenous treatment effect model, the predicted probabilities of the decisions to smoke and drink wine/beer calculated from the first stage (seemingly unrelated bivariate probit model) were adopted to control for endogeneity. The calculated predicted marginal probabilities served as proxies for smoking and frequent wine/beer drinking, and replaced the observed behavior in the body weight equation [35].

The second condition involved the heterogeneous effects of smoking, wine/beer drinking, and leisure time screen viewing behaviors across the BMI distribution. Individuals may respond differently to smoking, drinking, and leisure time screen activity, depending on their location in the BMI distribution. Ordinary least squares (OLS) specifications estimate the central tendency of the marginal effects of different covariates on the conditional mean of the BMI. However, these marginal effects may over- or understate the impact of the covariates at different points across the BMI distribution and provide insufficient information. Following recent studies in the field of nutrition and obesity research [61,67], we employed a quantile regression (QR) framework for the second stage in order to explore the heterogeneous associations across the different quantiles of the BMI distribution [61,63].

BMI was defined by a vector of sociodemographic characteristics, such as age, marital status, and area of residence, as well as indicators describing leisure time screen activity (i.e., TV viewing, computer gaming, chatting/social networking, computer usage) and the predicted probabilities of smoking status and frequent wine/beer drinking computed at the first stage. Because the residuals were excluded from the predicted values, they were supposed to be exogenous in body weight estimation [68].

The mathematical expressions for both the seemingly unrelated bivariate probit model and the quantile regression are provided in the Appendix A. For the sake of comparison, the OLS results without controlling for endogeneity issues are also exhibited. All analyses were conducted separately for men and women to investigate gender differences.

## 3. Results

Table 1 shows the distribution of the BMI in the ALLBUS sample, indicating that approximately 44% of participants were normal weight, 36% were considered overweight, and about 18% were identified as obese. An analytical description of the sample characteristics is provided in Table 2. In comparison with females, male participants were more likely to smoke (33.3% vs. 24.1%) and consume beer and wine almost daily (19.8% vs. 5.3%), as supported by the application of non-parametric tests. There was also an increasing likelihood of a higher BMI in men than women, indicating than men are at higher risk for overweight (26.667 vs. 25.516; *t*-test = 7.188, *p* < 0.01). With respect to leisure time screen viewing behaviors, over half of the respondents surfed the internet on a daily basis, and up to 40% were classified as prolonged TV viewers. The application of chi-square test also showed a statistically significant influence of gender on daily internet usage, with men engaging in recreational internet surfing more frequently than women (65.5% vs. 57.2%).

### 3.1. First Stage—Smoking Behavior and Frequent Wine/Beer Drinking

The estimation of the correlation coefficient *ρ* for the female subsample was highly significant, suggesting that unobservable factors that were positively related to smoking were also positively linked to frequent wine and beer consumption in women (*ρ* = 0.212, *p* < 0.01) (Table 3). This mutual dependence between the two decisions was also supported by the likelihood ratio (LR) test, in which the null hypothesis that the correlation coefficient of the error terms of smoking and drinking choices was equal to zero was rejected (chi-square(1) = 7.816, *p* < 0.01). Therefore, neglecting possible simultaneity between smoking and frequent wine/beer drinking may yield biased and inconsistent estimates in female participants.

Concerning leisure time screen viewing activity, prolonged TV viewing time augmented the propensity for smoking in both genders, and regular wine and beer drinking in males. Computer gaming was also positively correlated with smoking in our sample, whereas daily recreational internet usage and social networking were inversely associated with men’s smoking habits and women’s beer/wine drinking patterns, accordingly.

### 3.2. Second Stage—BMI Estimation

For comparison reasons, the BMI equation was estimated using both the QR and the OLS procedures. The OLS estimates are presented in Table 4 for both genders. In the QR specification, we estimated the coefficients at four quantiles, namely, the 25th, 50th, 75th, and 90th quantiles, utilizing the predicted probabilities for smoking and frequent wine/beer drinking to replace the observed behavior [35]. For the sake of interpretation, we presented the estimations at the selected percentiles, as the 25th percentile refers to normal weight individuals; the 50th percentile corresponds to slightly overweight participants; and the 75th and the 90th percentiles correspond to overweight and obese individuals, respectively. Table 5 and Table 6 display the estimations of the QR application for male and female participants, respectively.

The coefficients between the QR and OLS models were quite different. The conventional OLS regression resulted in a negative correlation between smoking and body weight in the male subsample (*γ* = −0.513, *p* < 0.05), and a statistically insignificant association between the two outcomes in female participants. On the contrary, the QR results revealed that smoking has a positive and highly significant influence on BMI in both genders and indicated that the associations might differ in magnitude and statistical significance across different percentiles. In particular, smoking behavior in males was linked with a BMI increase by 3.724, 3.098, 8.288, and 8.378 units in the 25th, 50th, 75th, and 90th quantiles, respectively. In female participants, cigarette smoking was also associated with a higher BMI at the 25th and the 75th quantiles of the distribution by 3.595 and 7.324 units, respectively. It seems that smoking coefficients increase substantially in size for both genders at higher points of the BMI distribution, implying that cigarette smoking may augment obesity risks and may be correlated with weight gain, especially in overweight and obese individuals.

With respect to wine and beer drinking, the direction and the magnitude of the associations differed, depending on which method was applied. OLS estimates showed an inverse association between daily wine/beer consumption and BMI in females, whereas no significant correlation was detected in the male subgroup. On the other hand, the QR model indicated a negative relationship in men, with a BMI decrease being more likely to be observed in normal weight frequent consumers. Thus, a daily consumption of beer/wine was found to reduce men’s BMI by 6.112 units at the 25th quantile.

Our findings showed that neglecting potential endogeneity problems may result in biased and inconsistent estimates of smoking and wine/beer drinking in the BMI equation. Furthermore, variations in the magnitude of the association and statistical power across different quantiles of the distribution provided evidence that the factors influencing body weight were heterogeneous across the BMI distribution. Thus, the two-stage equations system, as proposed before, was far more adequate to investigate body weight determinants compared with the conventional OLS procedure.

Leisure time screen viewing behaviors also revealed a diversity of factors that are linked to body weight. More specifically, prolonged TV viewing was found to be positively correlated with women’s BMI in both models. However, the QR application showed notable differences among quantiles. Extended television viewing contributed to a BMI increase at the 50th and the 75th quantiles of the BMI distribution in females by 1.110 and 1.240 units, respectively. The QR approach also stated a body weight increase by 1.427 units in slightly overweight women (50th quantile) who engage in computer gaming daily. Although the OLS model resulted in insignificant relationships between frequent chatting/social networking and body weight for both genders, the QR estimation indicated a statistically significant decrease in males’ BMI by 0.592 units at the 50th quantile. Daily recreational internet usage was negatively associated with the BMI of normal weight and slightly overweight women. Namely, leisure time internet surfers had a lower BMI by 0.603 and 0.738 units at the 25th and the 50th quantiles of the distribution, respectively.

## 4. Discussion

Expanding upon previous literature, this study showed that there is a reciprocal relationship between smoking participation and wine/beer drinking in the subsample of female participants, in which smokers were more likely to consumer wine and beer on a regular basis. Our findings supplement previous literature noting the interdependency between these outcomes, and underscore the necessity to model smoking and drinking behaviors simultaneously [35,59]. Regarding low-alcohol beverages’ consumption, daily beer and wine drinking was found to mainly influence normal weight men’s BMI. After controlling for potential endogeneity issues and heterogeneity in estimations, the two-stage equations system application showed that normal weight male drinkers were more likely to have a lower BMI.

Experimental studies on beer and wine drinking noted that light-to-moderate and frequent consumption had no effect in weight gain or significant changes in body weight indicators in both genders [13,69,70,71]. The negative correlation between BMI and beer-wine drinking in normal weight men, as supported from our findings, may be linked to the lifestyle factors of this specific consumer segment. For instance, normal weight men may be more health conscious and physically active, or choose to substitute alcohol for more energy dense foods in their diet. Although smoking and wine/beer drinking were found to be positively interrelated in women and female smokers had a higher BMI, frequent drinking was statistically insignificant in determining females’ BMI. This may have to do with the fact that a lower percentage of women reported frequent wine and beer consumption (5.3%) compared with the substantially higher percentage of female smokers (24.1%). Our data also lacked information on the amount of alcohol consumed. Further research should be conducted in order to test body weight differences across various drinking patterns.

Contrary to previous research resulting in inverse or insignificant relationships between cigarette consumption and body weight [32,33,35,72], our findings showed a positive association between smoking and BMI in both genders, after controlling for potential biases induced by endogeneity. Smoking influence was also heterogeneous across the BMI distribution and seemed to accelerate in the upper quantiles. Thus, overweight and obese smokers experienced a higher increase in their body weight than their normal weight counterparts. In comparison with women, smoking seemed to have a major impact on males’ body weight. This study challenges the widespread opinion that smokers weigh less and smoking may constitute an effective appetite and weight control strategy [27,55]. Thus, policy makers may proceed with antismoking policy without concerns of an inevitable body weight increase as an aftermath of smoking reduction. As smoking seems to be positively related to body weight, health interventions could be designed and developed under a more effective and holistic orientation in order to curb both cigarette consumption and obesity.

According to recent evidence, health risk factors usually co-occur or cluster together, implying that most individuals usually adopt more than one unhealthy behavior [73]. Therefore, smoking may comprise a link to other unhealthy behaviors, such as physical inactivity and poor dietary patterns [64], which in turn may lead to weight gain and the development of obesity [74]. Our findings also suggested that smokers were more likely to be heavy TV viewers and/or frequent computer game users than non-smokers. According to a growing body of evidence noting a significant relationship between recreational screen time and body weight [4,38,43], this study also sought links between leisure-time screen usage and BMI. Therefore, it could be assumed that smokers may have a higher probability for overweight and obesity because they are more prone to obesogenic lifestyles, such as prolonged screen viewing time.

Watching TV seems to contribute to body weight increase [4,37,38] by either displacing physical activity or by the consumption of high-calorie foods while watching TV [75,76]. Furthermore, recent research has shown that mentally passive sedentary behaviors, such as TV viewing, are positively associated with depressive symptoms [77,78] that may trigger overeating and lead to weight increase [56,79]. In addition, TV programs, such as cooking shows that often present food as a type of entertainment, may enhance food consumption [39], which in turn may result in subsequent weight gain. A comparison of TV viewing time between genders demonstrated that prolonged TV watching has a predominant impact on female participants’ BMI. Xie et al. [30] found a similar trend in the relationship between TV viewing time and body weight in the female subgroup and pointed out that women have a higher fat mass and a lower skeletal muscle on average, leading to differences between genders in the negative consequences of sedentary behavior [38]. Furthermore, when women allocate more time to watching TV, they may decrease physical activity. Previous studies have suggested gender differences when it comes to leisure time physical activity, indicating than men are more active than women [80,81]. Our findings also showed that prolonged TV viewing has a major influence on overweight women. Therefore, health interventions should primarily focus on specific consumer segments and orientate them towards alternative activities during leisure time in order to decrease watching TV. Special attention should also be paid to obesity prevention programs tailored to women, as they seem to spend more time in front of the TV [38].

This study also showed differences in the impact of recreational internet and video game usage on body weight, implying that the content of the usage seems to play a critical role in determining BMI status. In particular, recreational internet usage was found to decrease the BMI of normal weight and slightly overweight female participants, whereas involvement with social networking and chatting was inversely associated with the BMI of slightly overweight males. Contrary to our estimations, previous studies demonstrated either a strong correlation between recreational internet usage and overweight [44] or a rather insignificant relationship between them [43]. With respect to gender differences, women are more health-conscious and they are also more likely to seek for health-related information online [82]. Therefore, female participants may have increasing likelihood of obtaining information on health issues, and hence increase their knowledge on foods and nutrition, improve their eating habits, and become more oriented toward a healthy lifestyle.

On the other hand, playing computer games was more likely to lead to a body weight increase in slightly overweight women. Although there is a significant amount of research exploring the influence of recreational internet and computer usage on the BMI of children and adolescents, the number of studies that address this topic in the adult populations is limited. However, recent evidence also showed that regular computer gaming augments the probability for overweight [43,83]. One possible explanation is the sedentary nature of the activity and the time spent in front of a computer screen. In comparison with leisure time internet usage and chatting/social networking, the duration of sedentary bouts may be higher and the number of breaks may be lower in computer gaming. Gender differences on the impact of computer gaming and TV viewing on BMI may also be due to cultural influences that primarily encourage males’ participation in sports and physical activity [80], with women being more likely to substitute leisure time physical exercise with recreational screen time. Furthermore, women who spend more time in front of the computer or watching TV are also more likely to spend more time on other sedentary activities, resulting in even lower energy expenditure and body weight increase.

### Limitations

Despite the comprehensive methodological approach accounting for both endogeneity issues and heterogeneity of the main variables of interest, as well as the large sample of randomly selected participants, this study has some limitations that warrant consideration. Because of the cross-sectional design, we cannot draw conclusions about the direction of any causal relations between body weight and the main variables of interest (smoking, wine and beer drinking, and leisure-time screen activities). Further research adopting longitudinal designs is needed to explore issues of causality. In addition, the usage of self-reported anthropometric data may influence the estimations. Individuals may show the tendency to overestimate their height and underestimate their weight, leading to biases in BMI calculation [84]. Factors associated with social desirability and social norms to conform to a certain body ideal may affect individuals’ reporting of their actual weight and height [85]. Although recent evidence underpinned that BMI comprises a good proxy for body weight [27] and our methodological framework considered potential measurement errors (endogeneity), future research should examine more accurate body weight measures. The available data also combined wine and beer drinking and did not include information about the quantities consumed. Further research should be employed to encompass drinking amounts in order to better interpret our results. Further research should also distinguish between beer and wine drinking, and explore potential differences on their influence on BMI separately. Although both wine and beer are considered as low-alcohol beverages in comparison with spirits, there have been identified differences in the sociodemographic profile of beer and wine consumers [6], which could also be linked to differences on their impact on BMI.

## 5. Conclusions

Our findings showed that smoking and wine/beer drinking were positively interrelated in women. After considering endogeneity, cigarette smoking was found to be positively associated with body weight. Smoking impact was also heterogeneous across the BMI distribution in both genders and smokers’ BMI followed an increasing trend, especially in the upper quantiles of the distribution. This study contradicts the widespread belief that smokers have a lower body weight and smoking helps control body weight and appetite [27]. Therefore, obesity prevention interventions could be combined with smoking cessation policies in order to combat both outcomes simultaneously. Regarding wine and beer drinking, frequent consumption was found to mainly influence males’ body weight, showing a negative correlation with BMI in normal weight men. Our findings lend further credence to the existing literature, pointing out that TV viewing time and computer gaming have a strong link with weight increase, especially in overweight women. Leisure time internet usage and chatting/social networking were found to have a negative impact on the BMI of normal weight female and slightly overweight male participants, respectively. In addition, smoking behavior was also correlated with recreational screen time, especially prolonged TV viewing and computer gaming. Health policies could direct consumers toward the adoption of healthier lifestyle practices, and provide alternatives for recreational time in order to substitute screen activity and reduce sedentary time.

## Figures and Tables

**Table 1:** BMI distribution (N = 3472).

Mean	26.107
Standard deviation	4.714
Minimum	14.840
Maximum	54.080
**Percentiles (%)**	
1	17.938
2	18.510
5	19.790
10	20.760
15	21.560
20	22.280
25	22.880
30	23.460
35	24.000
40	24.460
45	24.910
46	25.000
50	25.460
55	25.910
60	26.490
65	27.100
70	27.760
75	28.600
80	29.430
82	30.034
85	30.590
90	32.080
95	34.990

**Table 2:** Sample characteristics.

Variables	Males (N_1_ = 1762)	Females (N_2_ = 1709)	*t*-Test	*p*-Value
BMI *	26.667 (0.101)	25.516 (0.125)	7.188	0.000
			**Chi-square**	***p*-value**
Smoker	586 (33.3%)	412 (24.1%)	35.717	0.000
Frequent wine/beer drinking	349 (19.8%)	91 (5.3%)	164.545	0.000
**Sociodemographic characteristics**				
***Age***			3.157	0.206
18–29 years old	308 (17.5%)	275 (16.1%)		
30–60 years old	938 (53.3%)	961 (56.3%)		
Older than 60 years	514 (29.2%)	472 (27.6%)		
***Marital status***			77.825	0.000
Married	1039 (59.0%)	959 (56.2%)		
Divorced/widowed	165 (9.4%)	331 (19.4%)		
Single	556 (31.6%)	417 (24.4%)		
***Area of residence***			2.660	0.264
Big city	564 (32.0%)	540 (31.6%)		
Town	490 (27.8%)	516 (30.2%)		
Village/Rural area	708 (40.2%)	652 (38.2%)		
***Education***			12.879	0.002
Secondary education	973 (55.3%)	1020 (59.8%)		
Post-secondary education	386 (21.9%)	381 (22.3%)		
Postgraduate studies	400 (22.7%)	306 (17.9%)		
***Frequent social interactions***	142 (8.1%)	126 (7.4%)	0.573	0.449
**Leisure time screen viewing behaviors**				
Daily internet usage	1154 (65.5%)	976(57.2%)	25.308	0.000
Chatting/social networks daily	412 (23.4%)	419(24.5%)	0.644	0.422
Playing games on computer daily	132 (7.5%)	119(7.0%)	0.350	0.554
High TV viewing time	709 (40.3%)	717(42.1%)	1.090	0.297

* mean, standard deviations in parentheses.

**Table 3:** Seemingly unrelated bivariate probit model estimates for smoking status and frequent wine and beer drinking.

Variables	Males		Females	
	Smoking Behavior	Frequent Wine/Beer Drinking	Smoking Behavior	Frequent Wine/Beer Drinking
Constant	−0.174	−1.557 ***	−0.545 ***	−2.126 ***
	(0.132)	(0.171)	(0.137)	(0.278)
***Socio-demographic characteristics* ^1^**				
30–60 years old	0.244 **	0.506 ***	0.123	0.340
	(0.110)	(0.152)	(0.114)	(0.260)
Over 60 years old	−0.700 ***	0.932***	−0.818 ***	0.960 ***
	(0.146)	(0.175)	(0.155)	(0.280)
Married	−0.141	0.126	−0.284 ***	0.061
	(0.089)	(0.103)	(0.096)	(0.171)
Divorced/widowed	0.211	0.038	0.020	−0.036
	(0.133)	(0.145)	(0.123)	(0.200)
Big city	0.036	−0.044	0.052	0.132
	(0.084)	(0.093)	(0.091)	(0.134)
Village/Rural area	−0.173 **	0.042	−0.028	−0.015
	(0.081)	(0.087)	(0.086)	(0.133)
Post-secondary education	−0.350 ***	-	−0.330 ***	-
	(0.085)		(0.091)	
Postgraduate studies	−0.488 ***	-	−0.478 ***	-
	(0.093)		(0.106)	
Socializing	0.392 ***	0.404 ***	0.311 **	−0.315
	(0.118)	(0.132)	(0.125)	(0.273)
***Leisure time screen viewing behaviors***				
Daily internet usage	−0.146 *	−0.057	0.015	0.030
	(0.086)	(0.085)	(0.090)	(0.126)
Chatting/social networks daily	0.085	−0.148	0.041	−0.360 *
	(0.092)	(0.116)	(0.096)	(0.190)
Playing games on computer daily	0.271 **	−0.180	0.520 ***	0.236
	(0.122)	(0.156)	(0.129)	(0.211)
High TV viewing time	0.229 ***	0.157 **	0.331 ***	−0.157
	(0.072)	(0.076)	(0.077)	(0.118)
				
*ρ*	0.053		0.212 ***	
	(0.049)		(0.074)	
**Log-Likelihood**	−1806.799		−1156.809	

Standard errors are given in parentheses. ^1^ Age: 18–29 years old (omitted variable), Marital status: single (omitted variable). Area of residence: town (omitted variable), Educational attainment: secondary education (omitted variable). * *p* < 0.1, ** *p* < 0.05, *** *p* < 0.01.

**Table 4:** OLS estimates for BMI.

Variables	BMI	
	Males	Females
Constant	25.234 ***	23.027 ***
	(0.412)	(0.487)
***Socio-demographic characteristics* ^1^**		
30–60 years old	1.478 ***	1.964 ***
	(0.343)	(0.412)
Over 60 years old	1.557 ***	2.282 ***
	(0.434)	(0.525)
Married	0.665 **	0.646 *
	(0.278)	(0.348)
Divorced/widowed	0.434	0.834 *
	(0.410)	(0.434)
Big city	−0.535 **	−0.850 ***
	(0.256)	(0.308)
Village/Rural area	0.043	0.230
	(0.244)	(0.294)
***Leisure time screen viewing behaviors***		
Daily internet usage	−0.195	−0.515 *
	(0.247)	(0.301)
Chatting/social networks daily	−0.187	0.209
	(0.288)	(0.344)
Playing games on computer daily	1.085 ***	1.781 ***
	(0.386)	(0.490)
High TV viewing time	0.486 **	1.619 ***
	(0.215)	(0.265)
***Smoking/drinking behaviors***		
Smoker	−0.513 **	−0.295
	(0.219)	(0.294)
Frequent wine/beer consumption	−0.102	−1.375 **
	(0.255)	(0.546)
		
**R-squared**	0.060	0.104

Standard errors are given in parentheses. ^1^ Age: 18–29 years old (omitted variable). Marital status: single (omitted variable). Area of residence: town (omitted variable). Educational attainment: secondary education (omitted variable). * *p* < 0.1, ** *p* < 0.05, *** *p* < 0.01.

**Table 5:** Quantile regression estimates for male participants’ BMI.

Variables	25th Quantile	50th Quantile	75th Quantile	90th Quantile
Constant	20.876 ***	23.140 ***	23.045 ***	26.263 ***
	(0.698)	(0.693)	(1.082)	(1.798)
***Socio-demographic characteristics* ^1^**				
30–60 years old	1.670 ***	1.875 ***	1.074	1.194
	(0.381)	(0.572)	(0.729)	(2.273)
Over 60 years old	3.586 ***	3.554 **	2.955	4.101
	(1.031)	(1.323)	(1.838)	(2.662)
Married	1.650 ***	1.002	1.251 **	0.454
	(0.391)	(0.420)	(0.588)	(0.880)
Divorced/widowed	0.792 **	0.247	0.112	−1.173
	(0.343)	(0.588)	(0.792)	(1.113)
Big city	−0.480 *	−0.232	−0.211	−0.596
	(0.270)	(0.286)	(0.395)	(0.807)
Village/Rural area	0.459 **	0.218	0.784 *	0.184
	(0.253)	(0.278)	(0.429)	(0.661)
***Leisure time screen viewing behaviors***				
Daily internet usage	0.090	0.043	0.238	1.024
	(0.285)	(0.251)	(0.445)	(0.712)
Chatting/social networks daily	−0.366	−0.592 *	−0.586	−0.565
	(0.300)	(0.347)	(0.538)	(0.864)
Playing games on computer daily	−0.003	0.886	0.512	1.855
	(0.467)	(0.603)	(0.761)	(1.193)
High TV viewing time	0.186	0.046	−0.432	0.683
	(0.225)	(0.335)	(0.462)	(0.626)
***Smoking/drinking behaviors***				
Smoking ^2^	3.724 ***	3.098 **	8.282 ***	8.378 ***
	(1.380)	(1.365)	(2.299)	(3.136)
Frequent wine/beer consumption ^2^	−6.112 *	−4.021	3.598	1.343
	(3.467)	(5.306)	(6.287)	(8.484)
				
R-squared	0.059	0.043 ^3^	0.042 ^3^	0.034 ^3^

Standard errors are given in parentheses. ^1^ Age: 18–29 years old (omitted variable). Marital status: single (omitted variable). Area of residence: town (omitted variable). Educational attainment: secondary education (omitted variable). * *p* < 0.1, ** *p* < 0.05, *** *p* < 0.01. ^2^ Predicted probabilities computed at the first stage, ^3^ pseudo R-squared.

**Table 6:** Quantile regression estimates for female participants’ BMI.

Variables	25th Quantile	50th Quantile	75th Quantile	90th Quantile
Constant	19.536 ***	21.263 ***	22.031 ***	27.027 ***
	(0.706)	(0.945)	(1.363)	(2.155)
***Socio-demographic characteristics*** ^1^				
30–60 years old	1.188 ***	1.573 ***	2.354 ***	3.563 ***
	(0.364)	(0.483)	(0.695)	(1.049)
Over 60 years old	3.384 **	2.851 *	3.577 *	5.211
	(1.367)	(1.712)	(1.990)	(3.530)
Married	1.328 ***	0.939 **	1.250 **	1.265
	(0.408)	(0.407)	(0.612)	(0.887)
Divorced/widowed	1.380 ***	0.765	1.339 **	0.800
	(0.493)	(0.522)	(0.598)	(1.060)
Big city	−0.386	−0.586	−0.540	−1.879 *
	(0.310)	(0.375)	(0.493)	(1.009)
Village/Rural area	0.601 **	0.555	0.521	−0.117
	(0.249)	(0.349)	(0.473)	(0.880)
***Leisure time screen viewing behaviors***				
Daily internet usage	−0.603 **	−0.738 *	−0.431	−0.385
	(0.290)	(0.380)	(0.536)	(0.792)
Chatting/social networks daily	−0.032	0.495	0.243	−0.671
	(0.361)	(0.508)	(0.581)	(1.110)
Playing games on computer daily	0.636	1.427 **	0.953	2.267
	(0.772)	(0.655)	(1.304)	(2.157)
High TV viewing time	0.271	1.110**	1.240 *	1.737
	(0.331)	(0.502)	(0.637)	(1.155)
***Smoking/drinking behaviors***				
Smoking ^2^	3.595 **	3.119	7.324 **	5.319
	(1.786)	(2.073)	(3.570)	(5.612)
Frequent wine/beer consumption ^2^	−11.961	0.184	7.988	−12.468
	(12.769)	(14.470)	(17.535)	(30.126)
				
R-squared	0.062	0.067 ^3^	0.068 ^3^	0.066 ^3^

Standard errors are given in parentheses. ^1^ Age: 18–29 years old (omitted variable). Marital status: single (omitted variable). Area of residence: town (omitted variable). Educational attainment: secondary education (omitted variable). * *p* < 0.1, ** *p* < 0.05, *** *p* < 0.01. ^2^ Predicted probabilities computed at the first stage, ^3^ pseudo R-squared.

## Data Availability

ALLBUS data can be provided by GESIS-Leibniz Institute for the Social Sciences.

## Abbreviations

BMI—body mass index; TV—television.

## Appendix A

***First stage: The seemingly unrelated bivariate probit model***

The seemingly unrelated bivariate probit model is described as follows [64,65]:

(A1) $$Y_{i1=}^{*}X_{i1}\beta_{1}+\varepsilon_{\iota 1},\;\;\;\;\;\;Y_{i1}=1\;\;\;\;if\;\;\;\;Y_{i1}^{*}>0,\;\;\;\;0\;otherwise$$

(A2) $$Y_{i2=}^{*}X_{i2}\beta_{2}+\varepsilon_{\iota 2},\;\;\;\;\;\;Y_{i2}=1\;\;\;\;if\;\;\;\;Y_{i2}^{*}>0,\;\;\;\;0\;otherwise$$

$$\begin{array}{c} E\left[\varepsilon_{i1}|{Χ}_{i1},{Χ}_{i2}\right]=Ε\left[\varepsilon_{i2}|{Χ}_{i1},{Χ}_{i2}\right]=0\\ Var\left[\varepsilon_{i1}|{Χ}_{i1},{Χ}_{i2}\right]=Var\left[\varepsilon_{i2}|{Χ}_{i1},{Χ}_{i2}\right]=1\\ Cov\left[\varepsilon_{i1},\varepsilon_{i2}|{Χ}_{i1}{Χ}_{i2}\right]=\rho \end{array}$$

where observations for smoking participation *Y_1_* and frequent wine/beer consumption *Y_2_* were available for the *i*th individual, $Y_{i1\;}^{*}$ and $Y_{i2\;}^{*}$ were the latent variables from which the decisions to smoke and drink were defined, and the stochastic errors *ε_i_*_1_ and *ε_i_*_2_ were assumed to be bivariate standard normal jointly distributed. The correlation coefficient *ρ* estimated the degree of simultaneity between smoking and wine/beer drinking, and in the case in which it was equal to zero, the two outcomes could be modelled independently by two univariate probit models [65]. The vector *X_i_*_1_ included all the explanatory variables affecting the decision to smoke, whereas the factors influencing wine and beer drinking were encompassed in vector *X_i_*_2._ The estimates of the vectors *β*_1_ and *β*_2_, and the correlation coefficient *ρ*, were obtained from the maximum likelihood function [65].

***Second stage: BMI estimation—quantile regression***

The QR technique is analytically described below [60,64]:

Let Wi indicate the BMI of the ith individual and Qθ(wi|Zi) for θ ∈ (0, 1) stand for the θth conditional quantile of the distribution of BMI (wi), given a vector Zi of k covariates. The QR model referring to the θth quantile can be expressed as follows:

W_i_ = **w**_θ_**Z**_i_ + ε_θi_ (A3)

Q_θ_(W_i_|Z_i_) = **w**_θ_**Z**_i_
(A4)

where *w_θ_* is a vector of the QR coefficients that depend on *θ*, Z_i_ is a vector of explanatory variables, and *ε_θi_* is the error term. The QR coefficients can be estimated by minimizing the objective function:

(A5) $$\;min_{w}\left[\sum_{i:\;W_{i}\geq wZ}\theta \left|W_{i}-w_{\theta }{Ζ}_{i}\right|+\sum_{i:\;W_{i}\leq wZ}\left(1-\theta \right)\left|W_{i}-w_{\theta }{Ζ}_{i}\right|\right]$$
